# The Anti-Nociception Effect of Dezocine in a Rat Neuropathic Pain Model

**Published:** 2014

**Authors:** Fei-Xiang Wu, Rui-Rui Pan, Wei-Feng Yu, Renyu Liu

**Affiliations:** 1 Department of Anesthesiology and Critical Care, Perelman School of Medicine at the University of Pennsylvania, Philadelphia PA 19104, USA; 2 Department of Anesthesiology, Eastern Hepatobiliary Hospital, Second Military Medical University, Shanghai 200438, China

## Abstract

The treatment of neuropathic pain (NP) currently remains clinically challenging. In an attempt to identify novel targets of known opioids, we found that dezocine, a non-addictive opioid, inhibits norepinephrine and serotonin reuptake through their transporter proteins which open the potential for dezocine to manage NP. In the present study, the effect of dezocine on NP was observed in a rat model of chronic constriction injury (CCI). The paw withdrawal latency (PWL) and paw withdrawal threshold (PWT) were used to evaluate thermal hyperalgesia and mechanic allodynia for nociceptive response. PWL and PWT tests were performed at 11:00 AM starting from 1 day before CCI surgery and 1, 3, 7, 10 days after right sciatic nerve ligation in the presence or absence of daily intraperitoneal injection of dezocine. The results demonstrated that the CCI-induced thermal and mechanical pain hypersensitivity was attenuated by dezocine significantly and persistently without sign of tolerance, indicating that dezocine could be an alternative medication for the treatment of NP. Clinical trial to confirm such discovery is warranted.

## Introduction

Neuropathic pain (NP), characterized by hyperalgesia and allodynia, is often due to nerve injuries in peripheral or central nerves system caused by trauma, inflammation or other diseases. [1] NP is affecting about 3–4.5% of the global populations and is being recognized as a major economic and social burden. [2] However, the pathophysiological mechanism of NP remains unclear and the treatment of NP is still a clinical challenge.

Opioid analgesics, such as morphine, are the widely used for various pain. But for neuropathic pain, studies have shown considerable controversy for opioid analgesics.[3] While opioids have been reported to be not effective for NP in some clinical studies [4,5] and opioids have notorious side effects (constipation, nausea, vomiting and sedation).[6,7] Efforts finding opioids as alternative medication for NP management never stop. Some recent reports indicate that some types of opioids or different route of opioid administration are effective in suppressing NP.[8,9] Buprenorphine for pain management is gaining popularity in recently years due to its unique pharmacological profile as partial mu agonist and kappa antagonist and its clinical role in addiction and NP treatment. However, buprenorphine itself can generate dependence and addiction for long-term usage,[10] which is a limiting factor for its clinical use for pain management.

Similar to buprenorphine, dezocine is also a partial mu agonist and kappa antagonist.[11] Dezocine also inhibited the norepinephrine transporter (NET) and serotonin transporter (SERT).[11] As transporters, NET/SERT respectively transport norepinephrine (NE)/serotonin into presynaptic neurons, both of which have been suggested to be associated with NP pain. [12–14] Most importantly, dezocine is not a controlled substance despite it is an opioid and has been wildly using for post-operative pain treatment.[15] Such unique pharmacological profile of dezocine indicates that it has a potential of reducing neuropathic pain.

In the current study, we hypothesized that dezocine could attenuate neuropathic pain and demonstrated such hypothesis using a rat neuropathic pain model of chronic constriction injury (CCI) to sciatic nerve in rats.

## Materials and Methods

Dezocine (5mg/1ml containing in an ampoule, Approval Number: H20080329) was obtained from Yangtze River Pharmaceutical Group (Jiangsu, China). Sodium pentobarbital was obtained from Chemical Reagent Company (Shanghai, China).

### Animals

The National Institute of Health guidelines for Ethical Conduct in the Care and Use of Animals were strictly followed the experimental protocol approved by the institutional review Committee of Experimental Animal Care. Male Sprague-Dawley rats (Age: 10–12week, Weight: 200–250g, from Shanghai Experimental Animal Center of Chinese Academy of Sciences) were housed in a specific pathogen free (SPF) environment with a 12/12 hour light/dark cycle.

### Chronic constriction injury (CCI) model

CCI procedures on the sciatic nerve of male SD rats were performed as previously described.[16] Briefly, after rats were anesthetized by i.p. injection of sodium pentobarbital (40 mg/kg), the right sciatic nerve of the mid-thigh level was exposed. Chromic gut 4–0 was loosely tied around the nerve for 4 ligatures with about 1 mm between knots. The ligation was performed to just barely reduce the diameter of sciatic nerve. The ligatures caused intraneural edema and resulted in constriction of nerve. In the sham group, the sciatic nerve was exposed without ligation. The incisions of rats were closed in layers. After recovery from anesthesia, rats were housed individually in the clear plastic cages with soft bedding covered with 3–6 cm of sawdust.

### Experimental Protocol

Rats were randomly assigned to three groups (6 rats in each group): a sham group (IP normal saline, IP NS), an NS group (CCI+ IP NS) and a Dezocine group (CCI+ IP dezocine). In the dezocine group, rats of CCI model received intraperitoneal (IP) injection of 3 mg/kg (in 2ml of volume) body weight of dezocine at 9:00 AM per day starting for the day of the surgery. Same volume of normal saline (2ml) was injected in the other two groups at the same time.

### Evaluation of thermal hyperalgesia

The paw withdrawal latency (PWL) to radiant heat was used to evaluate thermal hyperalgesia for nociceptive response as previously described.[17] Rats were placed in transparent plexiglass cage (23×18×13cm) with a piece of 3-mm-thick glass floor and received heat radiation after acclimating to the environments for 30 minutes. The radiant heat source consists of a high-intensity projection lamp bulb (8V, 50W), which was located 40 mm below the glass floor beneath the right hind paw of the rats. The heat source projected through a 5×10-mm aperture on the top of a movable case. A digital timer automatically measured the duration between the starting of heat and the paw withdrawal, which was considered as the PWL. The PWL was measured in 0.1 second and a maximum of 20 seconds exposure to radiation was set to avoid injury. Three repeated measurements were performed in each rat with a 5-minute interval between each measurement. PWL tests were performed at 11:00 AM starting from 1 day before CCI surgery and 1, 3, 7, 10 days after surgery.

### Evaluation of tactile allodynia

The paw withdrawal threshold (PWT) was used to evaluate mechanical allodynia for nociceptive response with Von Frey filaments. The rats were placed in transparent plexiglass cage with a wire mesh floor. After acclimating to their environments for 30 minutes, each filament was applied perpendicularly to the plantar surface of the right hind paw. The end point was determined as paw withdrawal accompanied by biting, head turning and/or licking. The force (in gram, g) needed for this reaction was recorded. [18] The PWT was taken though increasing and decreasing the stimulus strength sequentially with the ‘up-and-down’ method as described by Chaplan.[19] Similar to PWL test, PWT tests were performed at 1 day before and 1, 3, 7, 10 days after CCI surgery.

### Statistical analysis

All data were presented as mean±SEM. Statistical analysis was performed using two-way ANOVA via GraphPad Prism5 software (GraphPad Software Inc, CA, USA). P<0.05 was considered statistically significant.

## Results

**Fig. 1:**
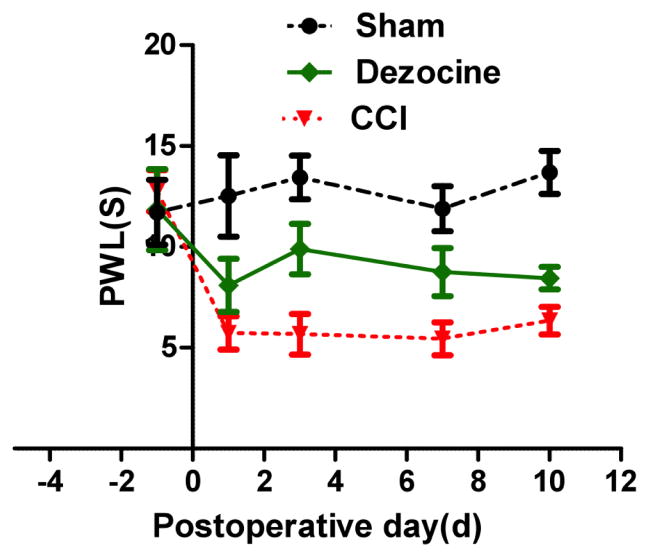
Changes of PWL after injection of dezocine on CCI rats Rats were administered with dezocine one day before CCI, and then PWL was measured. Following administration of dezocine, PWL was significantly increased comparing to that in the NS group (*P<0.05).

**Fig. 2:**
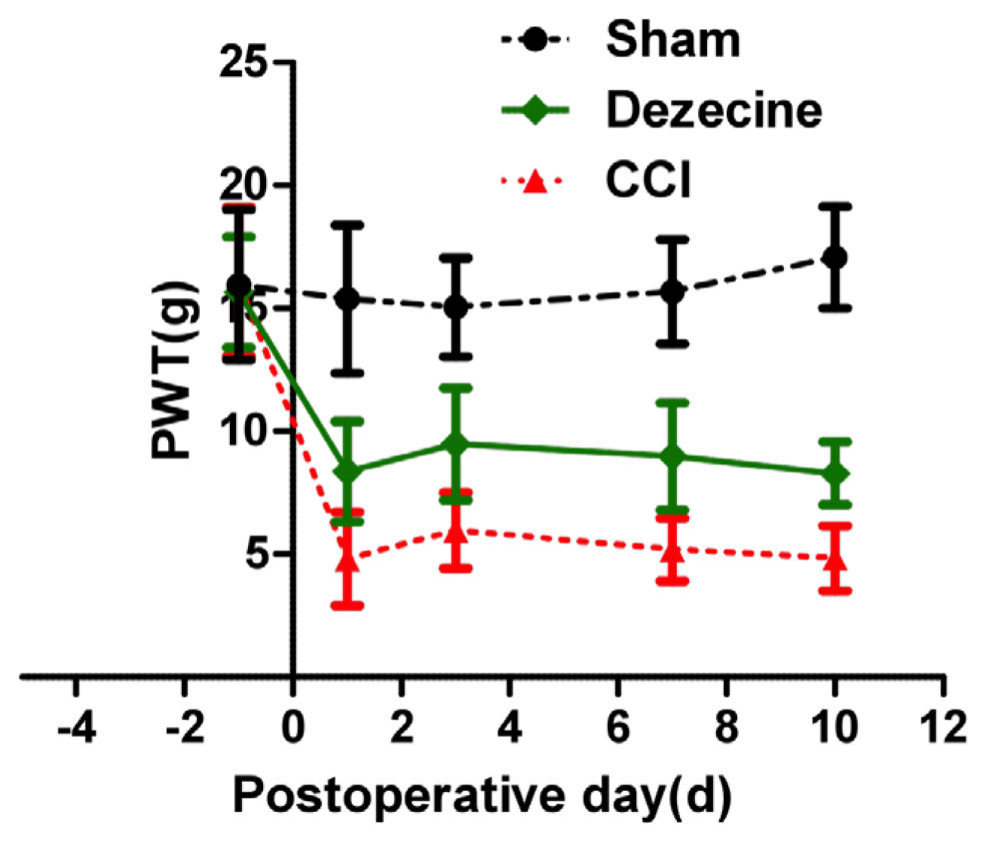
Changes of PWT after injection of dezocine on CCI rats PWT was monitored one day before CCI and 1, 3, 7, and 10 day after surgery. At 1st, 3rd 7th and 10th day, PWT showed markedly increased comparing to that in the NS group (*P<0.05).

After surgery, the PWL, representing the threshold of thermal hyperalgesia, decreased significantly compared to sham group. Statistically significant difference was found between the NS group and the sham group on 1, 3, 7, 10 days after CCI surgery (P<0.05, figure 1). Comparing to NS group, after dezocine administration, PWL significantly improved in the dezocine group lasting for 10 days without signs of fluctuation (P<0.05), suggesting that dezocine could attenuate thermal hyperalgesia during the whole experimental period without signs of tolerance. PWT was utilized to measure mechanical allodynia. Mechanical allodynia was induced by CCI, as evidenced by the reduction of PWT (figure 2). CCI rats receiving intraperitoneal injection of dezocine, PWT was increased markedly in the dezocine group comparing to the NS group (P<0.05), which suggested an attenuation of allodynia by dezocine (figure 2). Similar to PWL, the improvement of PWT was found during the entire experiment period. Taken together, the anti-nocicetion effect by dizocine started immediately after administration lasted for 10 days without signs of tolerance.

## Discussion

In the present study, the effect of dezocine on NP was investigated in a rat CCI model. The results indicated that dezocine significantly attenuated the CCI-induced thermal and mechanical pain hypersensitivity, indicating that dezocine could be an alternative medication for the treatment of NP. While this is a rather simple study, the novel clinical implication for dezocine is enormous due to lack to good medication for NP management.

Dezocine is a non-addiction analgesic drug with partial mu-opioid agonist and kappa-opioid antagonist activity,[11,20] which was used for alleviating post-operative pain.[21] The analgesic effect is approximately equipotent with morphine after administration of dezocine with therapeutic doses for moderate to severe pain.[22] The same analgesic effect of dezocine could reach that of fentanyl for postoperative pain treatment.[23] Partial mu-opioid agonist generally has fewer the side effects such as respiratory depression, tolerance, drug dependence and pruritus, than that of full mu-opioid agonist.[24,25] Tolerance and addiction serve as major barriers for the long-term usage of opioids for neuropathic pain especially for full mu-opioid agonist like morphine.[26] In the present study, no significant tolerance is observed for dezocine after intermittent injection of dezocine for consecutive 10 days. Tolerance generally develops after 7 days of morphine administration in rats.[27,28] Further studies are needed to confirm the finding of lack of tolerance for longer time period of usage.

While the mechanism of effectiveness of dezocine in reducing NP, it is possible that such therapeutic effectiveness may be related to the novel targets of dezocine we identified recently. In addition to opioid receptors, both NET and SERT were identified as novel targets of dezocine in our recent work using molecular target profiling. Dezocine inhibited norepinephrine and serotonin reuptake in a concentration-dependent manner in vitro. Through norepinephrine reuptake, NET plays a role in regulating the concentration of NE which is an important factor in descending inhibitory pathway.[29] In NP models of L5–L6 spinal nerve ligation, the increasing of NET density in the lumbar spinal cord was found, suggesting NET as a target for NP treatment.[30] Various studies have demonstrated the analgesic actions of NP through norepinephrine reuptake inhibitors.[31,32] SERT has proven to be involved in NP and serotonin reuptake inhibitors have indicated to be effective for NP treatment through inhibition of SERT. [14,33] It is important to know that many drugs such as duloxetine, amitriptyline, and Thien-2-yl 1S, 2R-milnacipran analogues, are all mixed NET/SERT re-uptake inhibitors, which have been shown efficacy against NP in different animal models and in clinical use.[34–37] In our present study, the attenuation effect of allodynia and hyperalgesia in NP model by intraperitoneal injection of dezocine was found. Based on the role of NET and SERT in NP and our previous founding that NET and SERT are new targets of dezocine, we assume that the anti-nociception effect of dezocine may through the inhibition of norepinephrine and serotonin reuptake. Thus, dezocine may be a good choice of NP treatment through opioid system and norepinephrine/serotonin system. Further studies are needed to confirm related mechanism and demonstrate the effectiveness in clinical trials.

## Conclusion

In summary, dezocine significantly attenuated the nociception effect in a neuropathic pain model in rats; indicating that dezocine could be an alternative medication for neuropathic pain management.
